# Removal of mercury from water by phytoremediation process with *Salvinia natans*(L.) All.

**DOI:** 10.1007/s11356-023-27533-w

**Published:** 2023-06-30

**Authors:** Magdalena Sitarska, Teodora Traczewska, Anna Hołtra, Dorota Zamorska-Wojdyła, Wiktoria Filarowska, Beata Hanus-Lorenz

**Affiliations:** 1 Faculty of Geoengineering, Mining and Geology, Wrocław University of Science and Technology, Wybrzeże S. Wyspiańskiego 27, Wrocław, 50-370 Lower Silesia Poland; 2 Faculty of Environmental Engineering, Wrocław University of Science and Technology, Wybrzeże S. Wyspiańskiego 27, Wrocław, 50-370 Lower Silesia Poland; 3 Department, US Pharmacia Sp. z o.o., Ziebicka 40, Wrocław, 50-507 Lower Silesia Poland

**Keywords:** Bioconcentration factor (BCF), Chlorophill a and b, Hg Pollution, Relative growth rate (RGR), Rhizofiltration, Total protein

## Abstract

Mercury contamination from human activities is a severe environmental problem. The low cost of rhizofiltration of heavy metal-contaminated environments is causing an increasing interest in these technologies. The present study demonstrates the effectiveness of mercury removal from water by phytoremediation using *S. natans*. Plants cultured and collected from the environment were used. The study used Hoagland’s liquid medium contaminated with mercury: 0.15, 0.20, and 0.30. The bioconcentration factor obtained was 275–780. The relative growth rate was up to 0.12 g/gd and was much better for cultured plants than those collected from the environment. The removal rate of toxic metal was up to 94%. Total protein increased for cultures plants by up to 84%, while it decreased by up to 30% for those taken from the environment. Total chlorophyll for cultured plants decreased by up to 54%, which could be due to the toxic effect of the metal.

## Introduction

Heavy metals in the environment are widespread in many regions of the world (Kocman et al. , 2013; United Nations Environment Programme , 2018). Notably, we should pay extra attention to Hg because it is a highly toxic element for the human nervous system, especially the brain. Especially dangerous are organic forms of mercury, methylmercury, and dimethylmercury. Mercury has no biological function in cells, and its excessive amount leads to death (Wang et al. , 2022).

The increased concentrations of heavy metals in ecosystems (soil, water, air) result from natural processes, such as volcanic eruptions and the weathering of rocks (Shah and Daverey , 2020). In addition, it contributes to that anthropogenic activity related to mining, industry, agriculture (fungicides), urbanization, and improper waste disposal (Garcia-Mercadoa et al. , 2017; Sarwar et al. , 2017; Zhou et al. , 2018; Schneider , 2021). Artisanal and small-scale gold mining (ASGM) pose a particular risk of mercury contamination. Global gold prices have tripled in the last ten years. As a result, gold mining in rural areas of the whole world has increased significantly (Veiga et al. , 2014; Garcia et al. , 2015). An estimated 16 million people are involved in this activity. Annual production is approximately 380–450 tonnes. The most common extraction of Au in ASGM is by combination with mercury. It consists of grinding the mineral (raw material) and mixing it with Hg. The gold forms an alloy with mercury by which they have separated from the unwanted matter. As a result of heating in an open vessel, the mercury has volatilized, and pure gold is obtained (Seccatore et al. , 2014). Gaseous forms of mercury in the atmosphere can stay for 0.5–1 year (Beckers and Rinklebe , 2017). Locally higher amounts of mercury in the air contaminate agricultural fields and water (Morgana et al. , 2021). It is undesirable near large populations. This phenomenon often occurs in South America, East Asia, and Southeast Asia (Ottesen et al. , 2013; Zhou et al. , 2018; Kengni and Mostert , 2022). In areas with a high concentration of mining activities, a significant part of agricultural land may be inaccessible due to pollution (Wang et al. , 2016; Li et al. , 2020).

Heavy metals such as Hg, Pb, Zn, Cd, and Fe are highly toxic pollutants that adversely affect the environment and the health and life of living organisms. Therefore, they should be monitored and removed from water and soils (Beckers and Rinklebe , 2017; Sarwar et al. , 2017; Hesami et al. , 2018; Deb et al. , 2020; Zahra et al. , 2020).

Among the methods of environmental cleanup, phytoremediation is very popular. Phytoremediation uses plants and associated microorganisms (rhizobacteria, endophytes, mycorrhizal fungi) Deb et al. (2020). This method also promotes the sustainable use of natural resources by ensuring adequate quality of waters and soils in changing global environmental conditions (Makarova et al. , 2022). The method’s capabilities rely on mitigating heavy metal toxicity in plants through their immune system. Plants use different detoxification pathways, including enzymatic, non-enzymatic antioxidant reactions, deposition of toxins in cell walls, vacuoles, and metabolically inactive tissues, and chelation by ligands (Lajayer et al. , 2019; Zahra et al. , 2020).

In the selection of plants for phytoremediation, ornamental plants are the best. They seem to be more economically viable and beautify the environment. They also do not present problems with their presence in the food/feed chain, like edible or medicinal plants. The phytotoxic effects of heavy metals on ornamental plants, manifested by chlorosis, necrosis, or reduced growth and flowering, are reduced through multiple detoxification pathways (Lajayer et al. , 2019).

Like other heavy metals, mercury can accumulate in live organisms, including plants. The amount of mercury accumulation in plants depends on its chemical speciation. Mercury dissolved in water, or its "active" fraction, is more bioavailable to bacteria and plants. The "inert" Hg fraction has lower mobility and bioavailability (Shahid et al. , 2020). Also, many biotic (e.g., Hg methylating/resistant bacteria) and abiotic (e.g., substrate composition) factors influence the process (Yin et al. , 2018; Zhao et al. , 2020). It is also necessary to choose the species of plants accordingly. Plants hyperaccumulators can accumulate 100–1000 times more heavy metals than other plants without apparent phytotoxic effects (Chamba et al. , 2017).

The increasing exposure of surface waters to pollutants, including heavy metals, increases the need for effective treatment technologies. The solution is aquatic phytofiltration based on multidirectional treatment. It exploits the ability of macrophytes to uptake and degrade pollutants. Various aquatic plants, including floating plants, e.g. *Lemna minor* L., *S. natans*, and *Eichhornia crassipes* (Mart.) Solms, are used in aquatic phytofiltration. *L. minor*, *E. crassipes* and *Pistia stratiotes* L. are generally used to eliminate heavy metal ions from water. Several scientific studies have shown high bioaccumulation of heavy metals in plant tissues (Pang et al. , 2023).

In the presented studies, an attempt is made to check the effectiveness of the phytoremediation process of mercury waters using *S. natans*, commonly known as floating fern. It belongs to the *Salviniaceae* family. In some parts of the world are often invasive species. In a few hours, they can overgrow entire bodies of water (completely covering the surface of the water surface), posing a threat to the fish living there. In Poland and other temperate climate countries, they are often plants threatened with extinction. In Poland, we have only *S. natans*, but in many African and American countries, adding several varieties are found (Gałka and Szmeja , 2013; Pietryka et al. , 2018).

## Materials and methods

### Plant material used in the study

*S. natans* is a widely distributed plant in Eurasia and North Africa. It has also been imported into North America but is considered invasive in many southern US states (Gałka and Szmeja , 2013). The species is found primarily in standing water. It occurs mainly in Lower Silesia in Poland, and its growing season is April-October (Zajac and Zajac , 2001; Gałka and Szmeja , 2013; Pietryka et al. , 2018).

**Fig. 1:**
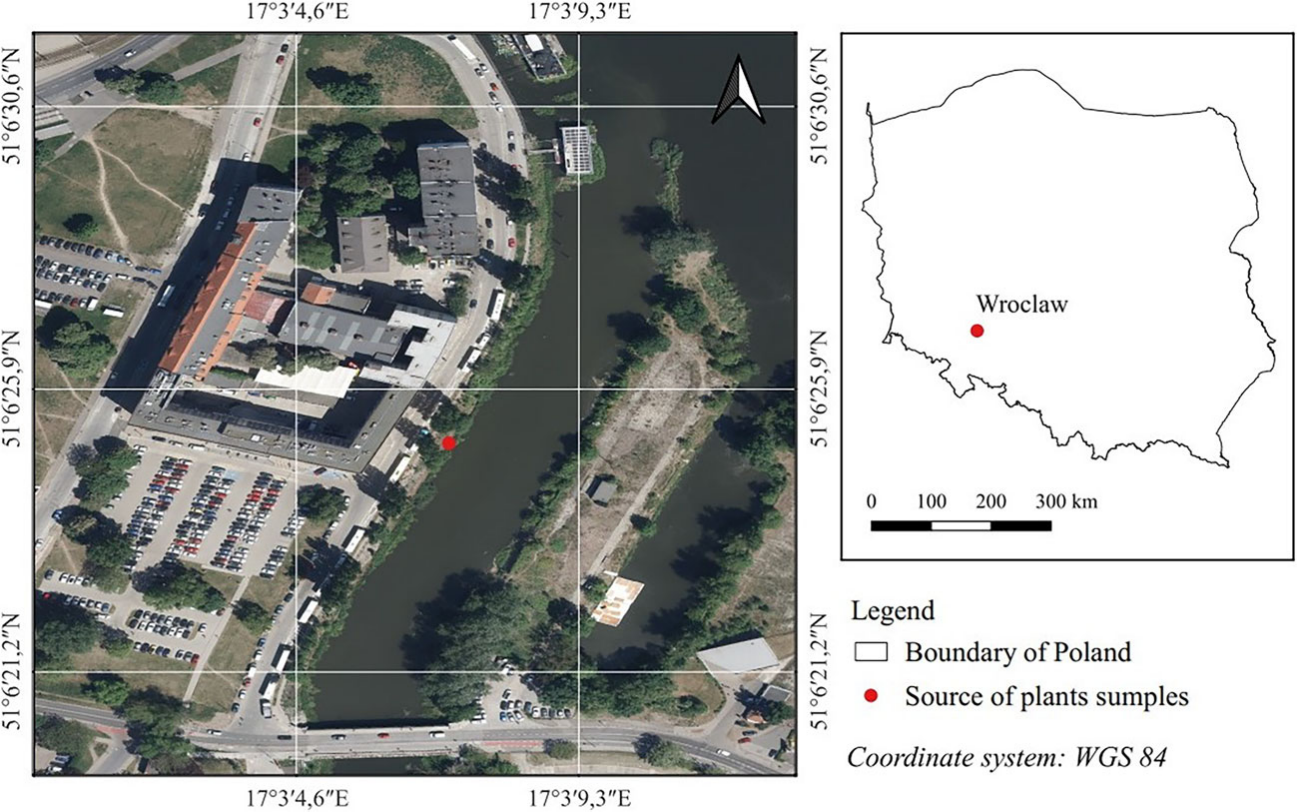
The geocoordinates of the sampling of plants

To assess the potential mercury phytoremediation depending on the origin of the biological material, the study on plants obtained from commercial breeding "cultured" (grown in artificial water tanks with a suitable substrate composition for plant growth) and taken from the natural environment. *S. natans* include on Red List of Plants and Fungi of Poland (Mirek , 2006). Before each collection of plants from the environment, we applied to the Regional Director of Environmental Protection for the possibility of collecting. Plants were taken from the Oława River (Fig. 1).

Upon completion of the collection, a relevant report was prepared and submitted to the Regional Director of Environmental Protection.

**Fig. 2:**
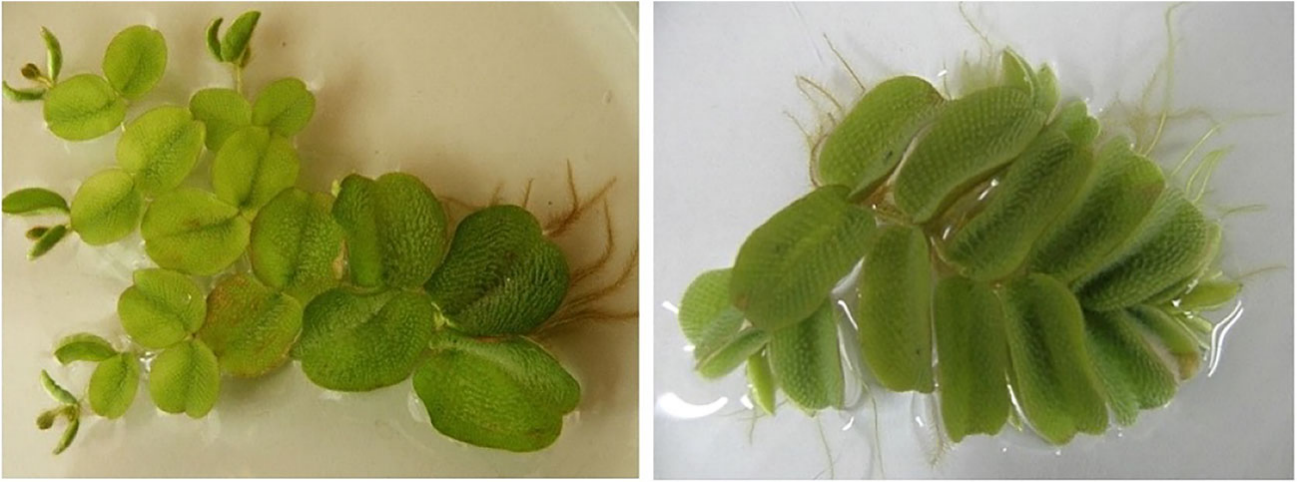
Plant morphology according to the origin (on the left cultured, on the right environmental)

The hydroponic experiment lasted 21 days. The liquid medium contained only the micro- and macronutrients necessary for the proper growth of aquatic plants. For each concentration of mercury in the liquid medium, 5 g of plants were used. The experiments were conducted under constant conditions using a Biosell FD 147 Inox phytotron, with a day/night cycle (12 h/12 h), the temperature of 22$\mathrm {^{o}}$C/15$\mathrm {^{o}}$C at 40% humidity Fig. 2.

### Preparation of the liquid culture medium

The study used a modified Hoagland’s medium (composition: KNO$\mathrm {_3}$ - 1.02 $\mathrm {g/dm^{3}}$, Ca(NO$\mathrm {_3}$)$\mathrm {_2}$×4 H$\mathrm {_2}$O - 0.71 $\mathrm {g/dm^{3}}$, NH$\mathrm {_4}$H$\mathrm {_2}$PO$\mathrm {_4}$ - 0.23 $\mathrm {g/dm^{3}}$, MgSO$\mathrm {_4}$×7 H$\mathrm {_2}$O - 0.49 $\mathrm {g/dm^{3}}$, MnCl$\mathrm {_2}$×4 H$\mathrm {_2}$O - 1.81 $\mathrm {mg/dm^{3}}$, H$\mathrm {_3}$BO$\mathrm {_3}$ - 2.86 $\mathrm {mg/dm^{3}}$, CuSO$\mathrm {_4}$×5 H$\mathrm {_2}$O - 0.08 $\mathrm {mg/dm^{3}}$, ZnSO$\mathrm {_4}$×7 H$\mathrm {_2}$O - 0.22 $\mathrm {mg/dm^{3}}$, FeSO$\mathrm {_4}$×7 H$\mathrm {_2}$O - 0.60 $\mathrm {mg/dm^{3}}$), which is a liquid medium characterized by a balanced content of micro and macronutrients necessary for the proper growth of aquatic plants (Hoagland and Arnold , 1950; Banerjee and Sarker , 1997; Zahra et al. , 2020).

In the case of mercury-contaminated solutions, Hoagland’s medium obtains the required concentration of an appropriate amount of mercury standard Mercury (II) nitrate standard of 1000 $\mathrm {mgHg/dm^{3}}$ for ASA from Merck (Certified reference materials - CRM).

### Selection of mercury concentrations

In order to select the concentration of mercury in the medium, which allows the phytoremediation process to be carried out without premature death of plants, preliminary studies were carried out. Hoagland media contaminated with mercury were prepared, obtaining solutions with its concentration: 0.035 mgHg/dm$\mathrm {^3}$, 0.075 mgHg/dm$\mathrm {^3}$, 0.150 mgHg/dm$\mathrm {^3}$, 0.200 mgHg/dm$\mathrm {^3}$, 0.300 mgHg/dm$\mathrm {^3}$, 0.350 mgHg/dm$\mathrm {^3}$, 0.450 mgHg/dm$\mathrm {^3}$, 0.500 mgHg/dm$\mathrm {^3}$.

For each concentration, 5 g of plants were placed in 500 $\mathrm {cm^{3}}$. The experiment lasted 14 days. Plant condition was assessed by visual observation of changes (chlorosis, necrosis) on the surface of plant leaves.

Based on the observations, the following concentrations were selected: 0.15 $\mathrm {mgHg/dm^{3}}$, 0.20 $\mathrm {mgHg/dm^{3}}$, 0.30 $\mathrm {mgHg/dm^{3}}$.

### Analyses of mercury in solutions and plant dry matter

Analyses of mercury concentrations in solution and plant dry matter were carried out using an Altem AMA 254 atomic absorption spectrometer (Száková et al. , 2004; Nowak et al. , 2013). In the method used to determine the amount of mercury in plant biomass, the test material did not require pretreatment of the samples, except for drying them at room temperature to air-dry form.

### Plant growth analysis

To control biomass grown was used the Radwag WAA 160/C/1 analytical balance. Plants were weighed before and after the experiment (fresh weight). After the experiments, plants were dried and weighed again.

Determination of plant dry weight before the experiment is not possible. A mathematical conversion of fresh plant biomass to dry weight was used. For this purpose, ten plant samples of 5 g. each was prepared. The plants were in different physiological condition. The samples were dried at room temperature and weighed.The averaged results were used in the conversions.

The formula for calculation of mean relative growth rate (RGR) was given by Fisher (1921) as follows:

$$\begin{aligned} \mathrm {RGR = \frac{lnW_{2}-lnW_{1}}{t_{2}-t_{1}}} \end{aligned}$$

where,

- $\mathrm {W_{1}}$ -initial dry weight of plant at time $\mathrm {t_1,}$
- $\mathrm {W_{2}}$ - final dry weight of plant at time $\mathrm {t_2}$,
- $$\mathrm {t_{1}}$$ - initial time,
- $$\mathrm {t_{2}}$$ - final time,

The RGR value was converted to a percent growth rate (PGR). Percent growth rate are used to compare plant growth performance and metal tolerance of plants in solution (Saengwilai et al. , 2017; Woraharn et al. , 2021).

$$\begin{aligned} \mathrm {PGR = \frac{W_{2}-W_{1}}{t}\cdot 100} \end{aligned}$$

where,

- $\mathrm {W_{1}}$ - initial dry weight of plant at time $\mathrm {t_1}$,
- $\mathrm {W_{2}}$ - final dry weight of plant at time $\mathrm {t_{2}}$,
- t - total days of plant growth,

### Metal content in plant samples and substratum

The bioconcentration factor (BCF) gives information about the plant’s ability to accumulate the target element. That is a parameter by which we can indirectly determine whether a plant has a high enough abiotic tolerance potential. When BCF$\le$1, the plant only absorbs the metal, if BCF>1, the ability to accumulate and stabilise the contaminant (Liu et al. , 2009; Sulaiman and Hamzah , 2018). The bioconcentration factor was obtained for the plants using equation (Lazo et al. , 2022; Makarova et al. , 2022).

$$\begin{aligned} \textrm{BFC} = \frac{\mathrm {metal\,concentration\;in\;plants}}{\mathrm {metal\;concentration\;in\; substrate}} \end{aligned}$$

As the accumulation of mercury in plants increases, it is removed from the substrate, which was measured using the removal efficiency (RE):

$$\begin{aligned} \mathrm {RE = \frac{C_i-C_f}{C_i}\cdot 100\%} \end{aligned}$$

where C$_i$ and C$_f$ were the initial and final concentrations of the element in the substratum (Lazo et al. , 2022).

### Analysis of plant tissue

Samples for the determination of total protein were hydrolysates obtained from plants. Chemical and physical denaturation was carried out to destroy the second-, third- and fourth-order structures in the proteins. The breaking of hydrogen bonds and thus increasing the accessibility to the determined in the Lowry method modified by Eggstein and Kreutz (Lowry et al. , 1951; Eggstein and Kreutz , 1955). The fresh plant was prepared by mechanical homogenisation of 0.1 g in 1 M NaOH solution. An Ultra-TurraxTube Driver homogeniser was used to homogenise the samples. The resulting homogeniser was then incubated in a water bath at 100$^o$C for 10 min (physical denaturation). After cooling, the hydrolysate was filtered on a soft filter to remove plant residues. The prepared solution was the sample for the determination of total protein in plant tissues. Absorbance was measured on a T80+ UV/VIS instrument at a wavelength of 750 nm. Based on the absorbance was reading the total protein from the standard curve.

We were a study in *S. natans* chlorophyll content before and after phytoremediation experiments. The chlorophyll content was analyzed using a spectrophotometer by the acetone extraction method (Su et al. , 2010). The amounts of chlorophyll a and b in the plants were determined by measuring absorbance in the extracts. Extracts were prepared by mechanically homogenising 0.1 g of fresh plant matter in 90% acetone. For the homogeninising using an Ultra-TurraxTube Driver homogeniser. The homogenate was extracted for 22 h in the dark and at 5$\mathrm {^{o}}$C. The next filtered through soft strainers. Absorbance measurements were made on a T80+ UV/VIS instrument at 663nm and 645 nm.

The concentrations of chlorophyll a, chlorophyll b, and total chlorophyll of Salvinia natans were calculated using the following equations (Kumar et al. , 2018):

$$\begin{aligned} \mathrm {total\;chlorophyll: 20.2 (A645) + 8.02 (A663)} \end{aligned}$$

$$\begin{aligned} \mathrm {chlorophyll\;a: 12.7 (A663) - 2.69 (A645)} \end{aligned}$$

$$\begin{aligned} \mathrm {chlorophyll\;b: 22.9 (A645) - 4.68 (A663)} \end{aligned}$$

## Results and discussion

The study was conducted under constant climatic conditions for both control and mercury-exposed samples (phytotron: temperature 22$\mathrm {^o}$C day/15$\mathrm {^o}$C night, humidity 40%).

In the study, were controlled changes content of mercury in the substrate and the biological material used for testing. Insignificant amounts of mercury were observed in control samples of the substrate in the range of 0.0003$-$0.0009 mgHg/dm$\mathrm {^3}$, for the initial days of the experiment. However, watched on test day 21 increase in mercury concentration up to 0.0210 mgHg/dm$\mathrm {^3}$ in the control samples. That could have been resulting in the increased amount of mercury in the air near the samples from the experiments. Mercury is an element with strong-volatile properties. The saturated vapor in air at 22$\mathrm {^o}$C is about 16 mg/m$\mathrm {^3}$ (Dumarey et al. , 2010). In commercially grown plants, the value of mercury ranged from 0.24$-$0.27 mg/g DM. In environmental plants, the levels of mercury were 0.25$-$0.80 mg/g DM. That may have been due to the presence of mercury in their habitat. Mercury concentration in rivers of the region of origin of plants is 0.0008–0.0014 mg/dm$\mathrm {^3}$ (Barej et al. , 2009).

Plants used in phytoremediation should be characterized by tolerance to high concentrations of contaminants, a high rate of accumulation or biodegradation, rapid growth, and high biomass production. Plants can accumulate pollutants by incorporating them into the structure of their cells or metabolize toxins as a natural effect of adapting to the harsh conditions of living in a contaminated environment (Hesami et al. , 2018; Lajayer et al. , 2019). Therefore, the effectiveness of the phytoremediation process conducted by plants is significantly influenced by their growth as measured by per cent growth rate and relative growth rate (Mustafa and Hayder , 2021; Ustiatik et al. , 2022). Many plants show the ability to hyperaccumulate mercury (Chamba et al. , 2017).

**Fig. 3:**
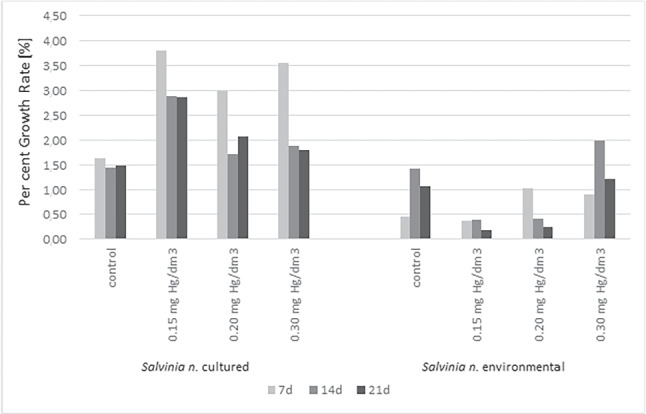
The per cent growth rate for *S. natans* cultured and collected from the environment

**Fig. 4:**
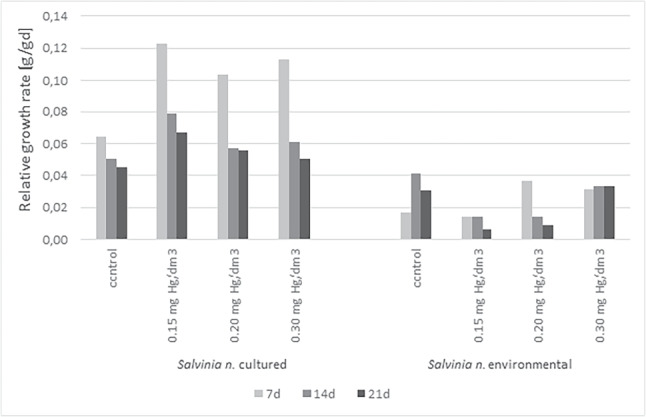
The relative growth rate for *S. natans* cultured and collected from the environment

The results, percent and relative growth rates, for cultured and environmentally sampled plants were shown in Figs. 3 and 4. The tests performed on cultured *S. natans* observed an increase in per cent growth rate in the range of 1.44$-$1.64% for controls and 1.72$-$3.82% for samples with mercury. The maximum increase was observed in samples with 0.15 mgHg/dm$\mathrm {^3}$ mercury, as much as 3.82% for day 7 and 2.89% for days 14 and 21. Also the samples with 0.2 mgHg/dm$\mathrm {^3}$ mercury, the percentage increase was higher than in the control samples. On day 7, it was 3.00%, and on day 14, it dropped to 1.72% on day 21, it increased again, reaching a value of 2.09%. For samples from a concentration of 0.3 mg Hg/dm$\mathrm {^3}$, a significant increase in PGR was also observed (7d$-$3.55%, 14d$-$1.90%,21d$-$1.80%) compared to the control sample (1.64%). This situation may have resulted from the stimulation of plants by the toxin, in that case, mercury is present in environmental (Sawidis et al. , 2018).

The biomass growth of plants is a valid macro-indicator (indicator) of their tolerance to environmental factors. Stunted growth and reduced biomass production were frequently observed in plants exposed to toxic levels of mercury (Xun et al. , 2017). Comparing the PGR results of control samples of commercial and environmental plants, there is less growth in plants taken from the environment. While the cultured plants scored 1.64% on 7d, the environmental plants only scored 0.45%. Only on days 14 and 21 did they achieve growth similar to the cultured ones, scoring 1.09$-$1.43%. Despite their earlier adaptation, was observed a decrease in the physiological condition of plants downloaded from the environment. It could have been a result of the stress associated with the change in growing conditions: transfer to a synthetic liquid medium (Hoagland’s medium), use of artificial lighting, and different light cycles. The adaptation period could be too short for the plants, so they showed the effects of severe stress (Zhang et al. , 2020).

Compared to the PGR results obtained for samples from mercury-contaminated solutions, significant differences are evident between the two biological materials. At the concentration of 0.15 mgHg/dm$\mathrm {^3}$ was observed a reduction in PGR for the total experiment compared to the control. That could have been the result of further stress by changing culture conditions, but also the appearance of the toxin (Zhang et al. , 2020). For concentrations of 0.2 mgHg/dm$\mathrm {^3}$ and 0.3 mg Hg/dm$\mathrm {^3}$, an increase in PGR was observed for the first seven days. It is possible that, in this case, the higher concentration of the toxin caused activation of its detoxification processes in plants or accumulation in tissues. At the concentration of 0.3 mgHg/dm$\mathrm {^3}$, this condition persisted until the 21st day. The PGR achieved values of 1.99% for 14d and 1.22% for the 21st day. However, for a concentration of 0.2 mgHg/dm$\mathrm {^3}$, it dropped to 0.42% and 0.25% on days 14 and 21, respectively. That suggests higher concentrations of the toxin trigger numerous defense processes to stimulate the growth of plants.

The RGR of cultured *S. natans* for all mercury concentrations was higher than the control samples. While the range for the control was 0.05$-$0.06 g/gd, in the mercury samples was 0.05$-$0.012 g/gd. The highest increase was observed on the 7th day of the experiment for all concentrations of mercury tested. The following days of the experimentation (14d, 21d) show a significant decrease in the RGR for the three tested concentrations compared to the 7th day. Most likely, by the appearance of the toxin in the substrate, the plants accumulated it, activating biomass growth processes. After the 7th day of exposure to the pollution, mercury accumulation in plant tissues began adversely affecting its metabolic processes. The weakest biomass growth at the highest concentration of the tested metal suggests the manifestation of the gradual effect of the negative impact of Hg ions (Sawidis et al. , 2018). The consequence was a significant slowdown in biomass growth.

**Fig. 5:**
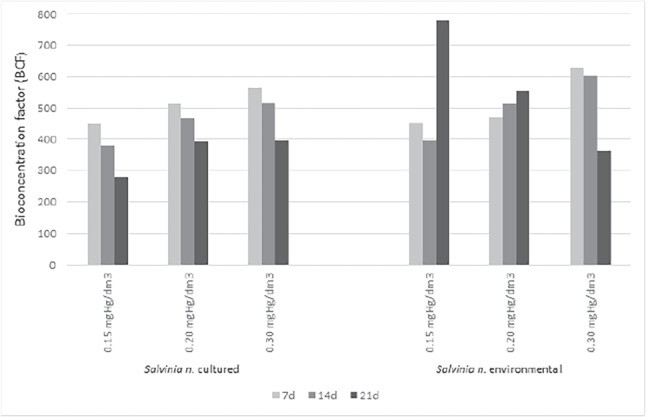
Bioconcentration factor for *S. natans* cultured and collected from the environment

The bioconcentration factor for cultured plant’s decrease was observed on successive days of plant exposure to mercury for all concentrations tested (Fig. 5). The BCF on the 7th day was 449, while on the 14th day, it decreased by 15% for solutions of 0.15 mgHg/dm$\mathrm {^3}$. The following days also showed a decrease which was 38% relative to the 7th day. In the study for solutions of 0.2 mgHg/dm$\mathrm {^3}$ was observed similar decreases on days 14 and 21 compared to day 7. The BCF on day 7th was 512, on day 14 was 468, and on day 21 was 393. The represents decreases relative to day 7 of 9% and 23%. The following concentration tested was 0.3 mgHg/dm$\mathrm {^3}$, which on day 7, the BCF was 567, on day 14d was 517 (a decrease of 0 9%), and on day 21 was 396 (decrease of 30% relative to day 7). The decreasing bioconcentration factor may be the release of mercury from dying plants into the solution.

Different bioconcentration trend was observed for plants taken from the environment compared to plants cultured. Solution of 0.15 mgHg/dm$\mathrm {^3}$ and 0.20 mgHg/dm$\mathrm {^3}$, the bioconcentration factor increased with the duration of mercury exposure. Only the concentration of 0.30 mgHg/dm$\mathrm {^3}$ was the highest bioconcentration observed on 7d, where the BCF was 630. On subsequent days, it decreased to 604 (14d) and 362 (21d). The highest bioaccumulation was observed on day 21 for a concentration of 0.15 mgHg/dm3, where the BCF was 780. An increase of 73% compared to day 7 (0.15 mgHg/dm$\mathrm {^3}$). It is significant that as the mercury concentration in the substrate increased, the BCF for each solution tested increased, despite the decreasing relative growth rate. That may suggest that plants can accumulate a significant amount of the toxin despite reduced biomass growth. The number of mercury in the substrate is a parameter that has a superior influence on the degree of accumulation than RGR.

Xun et al. investigated the ability of the plant species *Cyrtomium macrophyllum* (Makino) Tagawa to extract mercury from a contaminated mining area. The mercury concentration in the plants reached 36 mg/kg with a translocation factor of 2.62. The leaf tissue of *C. macrophyllum* showed high resistance to mercury stress (Xun et al. , 2017).

Zhao’s study showed that the amount of accumulation depends on the growth phase of the plants. The lowest accumulation is in the early stages of plant growth (Zhao et al. , 2019).

The higher accumulation in plants taken from the environment may be due to the presence of microorganisms that assist in mercury oxidation processes. Biosurfactants are metabolites produced by microorganisms, mainly by bacteria such as *Pseudomonas aeruginosa*, *Bacillus subtilis*, and *Lactobacillus sp.* These organisms assist in the desorption of toxic heavy metals ions, by improving their bioavailability to plants (Shah and Daverey , 2020). Endophytic bacteria supporting mercury phytoremediation are still little understood. Mello and others isolated 34 strains of epiphytic bacteria from the environment. To study the support of mercury phytoremediation, they selected 8 of them (*Acinetobacter baumannii* BacI43, *Bacillus sp.* BacI34, *Enterobacter sp.* BacI14, *Klebsiella pneumoniae* BacI20, *Pantoea sp.* BacI23, *Pseudomonas sp.* BacI7, *Pseudomonas sp.* BacI38 and *Serratia marcescens* BacI56) the most resistant to metal contamination. Except for BacI20, the remaining strains increased the growth of the bioaccumulator (maize) in a mercury-contaminated medium. Plants induced with BacI43 and BacI34 strains increased total dry biomass by about 47% (Mello et al. , 2020). The bacteria likely led to the remediation (volatilization) process, reducing the amount of metal in the substrate.

**Fig. 6:**
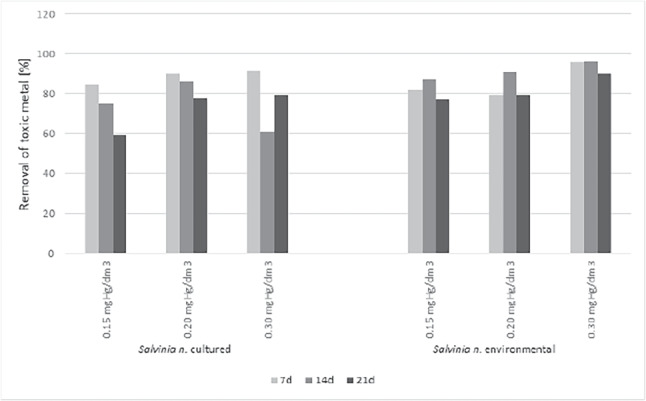
Removal of mercury from the substrate

A mercury accumulation study was conducted on different genotypes of Indian mustard grown hydroponically at five different Hg concentrations (0-50 $\mu$M). The genotype Pusa Jai Kisan was identified as the most Hg tolerant. It accumulated 269.9 $\mu$gHg/gDW in the underground parts and 61.7 $\mu$ gHg/gDW in the aboveground parts. It should be noted that ’Pusa Jai Kisan’ was developed using tissue culture techniques and is a somaclonal variety (Ansari et al. , 2021).

Phytoremediation processes are often carried out in special tanks, from which excess biomass can be easily harvested. The biggest problem of the phytoremediation process is the remaining significant amount of plant biomass, significantly contaminated with heavy metals. When designing this type of treatment plant, it is necessary to consider the management of the resulting biomass after the operation period. The most commonly used processes are composting, pyrolysis and compression landfill (Liu and Tran , 2021).

Accumulation of mercury in plant biomass removed it from solutions (Fig. 6). The mercury was removed from the substrate to the greatest extent during the first seven days of exposure for breeding plants. Solution of 0.15 mgHg/dm3, the removal of toxic metal was 85% after 7th days, for 14th days of exposure 75% and 59% for the 21st day. A similar relationship was found for concentrations of 0.20 mgHg/dm$\mathrm {^3}$ (7d-90%, 14d-86%, 21d-78%) and 0.30 mgHg/dm$\mathrm {^3}$ (7d-91%, 14d-61%, 21d-79%).

Gomes et al. investigated the effectiveness of mercury removal from water by phytoremediation. They used the macrophyte *Typha domingensis* Pers. in their study. They obtained a reduction efficiency of 99.6 ± 0.4% for mercury in contaminated water, with a mercury accumulation in the plant of 273.3515 ± 0.7234 mg/kg (Gomes et al. , 2014).

The effectiveness and low cost of the water phytoremediation process have contributed to their modification to increase process speed. Prasetya and others investigated mercury removal using subsurface flow constructed wetland (SSF-CW) composed of plants and natural zeolite. The method involved a combination of adsorption and phytoremediation mechanisms. Mercury-contaminated in the water at 14.94 mg/dm$\mathrm {^3}$ was pumped into the SSF-CW. After the experiment, the number of mercury in the test solution decreased by 91.84% (Prasetya et al. , 2020).

Arshadi and others studied the removal of lead (II) and mercury (II) from water using the floating aquatic fern *Azolla filiculoides* Lam. as a nanobioadsorbent element. The modified plants were coated with zero-valent nano-iron (NZVI) and then tested for the potential to adsorb/reduce Pb(II) and Hg(II) ions from aqueous media. Removal from the water of the toxic metals was observed after just 20 min. The plant inhabits water bodies in temperate and tropical climates of the Americas, Asia, and Australia. What gives it a wide range of possibilities for the treatment of water bodies in areas with small-scale gold mining (Arshadi et al. , 2017).

Garcia-Mercado and co-workers study mercury removal from soils taken from the sites of two closed mercury mines. The soils contained 424±29 mgHg/kg and 433±12 mgHg/kg.They used *Typha* L. and *Phragmites australis* (Cav.) Trin. ex Steud. plants for the phytoremediation process. They obtained reductions in mercury in the soil of 55–71% and 70–82% for *Typha* L. and 76–82% and 58–66% for *P.australis*, respectively.

Since the degree of mercury removal from the Hoagland’s medium decreased with the increasing duration of the experiment, this suggests a release of mercury from dying plants. That confirmed mercury studies in plant biomass, where we observed a decrease at the BCF.

Plants from the environment compared view cultured *S. natans* did not show the superior removal of mercury from the substrate during the first days of exposure. The highest removal values of the toxic metal were observed on the 14th day for all three solutions tested. The obtained results have not coincided with the BCF values, which did not reach a maximum value on the fourteenth day.

**Fig. 7:**
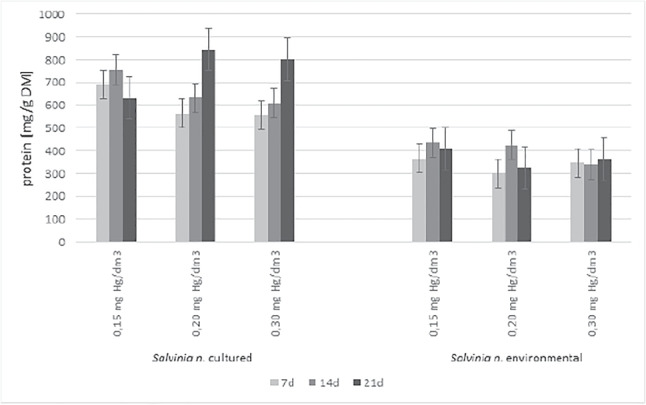
Total protein in *S. natans* cultured and collected from the environmental

**Table 1:** Chlorophyll for *Salvinia natans* cultured

concentration	time	chlorophyll a	chlorophyll b	chlorophyll	total chlorophyll
(mgHg/dm$\mathrm {^3}$)	(d)	(mg/g DM)	(mg/g DM)	a/b	(mg/g DM)
control	-	20.88±0.37	7.01±0.13	2.98±0.00	27.89±0.50
0.15	7	16.64±0.78	5.83±0.27	2.85±0.00	22.46±1.06
0.15	14	19.86±3.21	7.28±1.17	2.73±0.00	27.13±4.38
0.15	21	16.14±0.27	6.01±0.10	2.69±0.00	22.14±0.37
0.20	7	16.78±0.91	5.97±0.32	2.81±0.00	22.75±1.23
0.20	14	9.33±0.10	3.50±0.04	2.66±0.00	12.83±0.13
0.20	21	20.55±0.36	7.37±0.13	2.79±0.00	27.91±0.49
0.30	7	14.58±3.16	5.06±1.10	2.88±0.00	19.64±4.26
0.30	14	11.79±0.33	4.49±0.13	2.63±0.00	16.28±0.45
0.30	21	20.48±1.20	7.05±0.41	2.91±0.00	27.52±1.62

Source: Results of own research

The solution of 0.15 mgHg/dm$\mathrm {^3}$ was 381, and for a concentration of 0.2 mgHg/dm$\mathrm {^3}$ the BCF was 468. For the concentration of 0.3 mgHg/dm$\mathrm {^3}$, the BCF on day 14th decreased compared to day 7th. The next stage of the study was to assess the effect of accumulated mercury on plant physiological processes by measuring the amount of total protein (Fig. 7) and assimilation pigments (Tables 1 and 2). The total protein is responsible for biomass growth. Chlorophylls are involved in photosynthesis, the basic metabolic process of plants. Both parameters are important factors in assessing their physiological condition following exposure to the toxin.

In control samples of cultured *S. natans*, the amount of total protein averaged 457.64 mg/g DM. For the concentration of 0.15 mgHg/dm$\mathrm {^3}$, the total protein on day 7 was 51% higher than in the control samples, amounting to 690.87 mg/g DM. On day 14, it increased by another 15%, reaching a value of 755.26 mg/g DM. However, on day 21, the amount of protein decreased to 630.48 mg/g DM, but this was still higher than the control. Concentrations of 0.20 mgHg/dm$\mathrm {^3}$ and 0.30 mgHg/dm$\mathrm {^3}$, the amount of total protein in the plants were observed an increase on subsequent days of the experiment. On the 7th day, with a concentration of 0.20 mgHg/dm$\mathrm {^3}$, the amount of protein was 562.87 mg/g DM. On the 14th day, it increased by 38% relative to the control and was 632.15 mg/g DM, and on the 21st day by a further 47% obtaining a value of 844.47 mg/g DM. Comparable values were obtained for the concentration of 0.30 mgHg/dm$\mathrm {^3}$. On subsequent days of the experiment, the following values were recorded: 555.86 mg/g DM, 609.16 mg/g DM, and 802.08 mg/g DM.

For the total protein content of *S. natans* plant tissues taken from the environment, a significant reduction was observed on the first 7th day of exposure of the plants to mercury compared to the control. In the control samples, the amount of protein was 429.96 mg/g DM. For a concentration of 0.15 mgHg/dm$\mathrm {^3}$ on day 7, its amount was 366.51 mg/g DM and was 15% lower than the control.

On the 14 and 21 days, similar amounts were observed at 434.23 mg/g DM (14d) and 409.14 mg/g DM (21d). When tested for a concentration of 0.20 mgHg/dm$\mathrm {^3}$ on day 7th, the total protein was 300.08 mg/g DM, increasing by 30% lower than the control. On day 14, its amount was close to the control value at 425.43 mg/g DM, but on the 21st day, it decreased again relative to the control, by 24%, reaching a value of 325.27 mg/g DM. For a concentration of 0.30 mgHg/dm$\mathrm {^3}$, the amount of total protein was 347.35 mg/g DM on day 7, 340.12 mg/g DM on day 14, and 365.03 mg/g DM on day 21. The average amount of protein in plants in the presence of mercury was 18% lower than the control. The amount of total protein in plants in the presence of mercury at 0.30 mgHg/dm3 was comparable in all test periods. The changes did not change significantly with increasing exposure time.

Chlorophyll content in plants is the key factor for the photosynthesis process and growth and is also an important index of abiotic tolerance organisms of plants (Li et al. , 2016). In the study was controlled the changes of chlorophyll a and b. It was to assess the impact of mercury on plant health. In this aim, we also made mathematical conversions by determining the ratio of chlorophyll a to b and the amount of total chlorophyll.

**Table 2:** Chlorophyll for *Salvinia natans* collected from the environment

concentration	time	chlorophyll a	chlorophyll b	chlorophyll	total chlorophyll
(mgHg/dm$\mathrm {^3}$)	(d)	(mg/g DM)	(mg/g DM)	a/b	(mg/g DM)
control	-	8.42±0.65	2.83±0.22	2.98±0.00	11.24±0.87
0.15	7	2.54±0.06	0.81±0.02	3.14±0.00	3.35±0.08
0.15	14	6.56±0.01	2.39±0.01	2.75±0.00	8.95±0.02
0.15	21	6.21±0.04	2.23±0.01	2.79±0.00	8.43±0.06
0.20	7	7.02±0.05	2.86±0.02	2.46±0.00	9.88±0.06
0.20	14	3.58±0.00	1.45±0.00	2.46±0.00	5.04±0.00
0.20	21	7.97±0.46	2.97±0.17	2.68±0.00	10.94±0.63
0.30	7	10.87±0.96	3.68±0.32	2.95±0.00	14.55±1.28
0.30	14	3.34±0.18	1.34±0.07	2.48±0.00	4.68±0.26
0.30	21	6.44±0.23	3.89±0.14	1.66±0.00	10.33±0.37

Source: Results of own research

The cultured *S. natans* had more than 2.5 times the amount of chlorophyll a (20.88 mg/g DM) and b (7.01 mg/g DM) compared to the plants taken from the environment (chlorophyll a: 8.42 mg/g DM, chlorophyll b: 2.83 mg/g DM). However, in both cases, the chlorophyll a/b was close to 3.00. The plants usually have a ratio of chlorophyll a/b around 3:1. Its variability depends on the light tolerance and the age of the plants. P (Arnon , 1949; Rajalakshmi and Banu , 2015). Cultivated plants showed a decrease in chlorophyll a and b of about 17–19% in the first seven days of mercury exposure. Whereas for a concentration of 0.30 mgHg/dm$\mathrm {^3}$, the number of chlorophyll a was decreased by about 30%.

Similar results inhibited photosynthesis by reducing chlorophyll in plants were obtained by Zhao and others. At lower mercury concentrations, the amount of chlorophyll decreased by 43% after 60 days of mercury exposure. With increasing exposure time, the chlorophyll decreases in plants were no longer as great and did not exceed 10% relative to the control (Zhao et al. , 2019).

The quantitative chlorophyll a/b was close to 3:1 throughout the study period. The lowest value of 2.63 was observed on day 14 for a concentration of 0.30 mgHg/dm$\mathrm {^3}$. Please note chlorophyll a and b for concentrations of 0.20 mgHg/dm$\mathrm {^3}$ and 0.30 mgHg/dm$\mathrm {^3}$, which on day 14, have decreased almost halved. By day 21, however, their amounts were already close to the baseline values (control sample). It suggests that after the first few days of stress caused by the presence of the toxin, the plant returned to its total chlorophyll synthesis capacity (Beauvais-Flück et al. , 2018).

We observed a continuous decrease in chlorophyll a and b in the plants taken from the environment relative to the control. The most decrease was recorded on day 7th at a concentration of 0.15 mgHg/dm$\mathrm {^3}$. It was then 70% for chlorophyll a and b. On day 14, with a concentration of 0.20 mgHg/dm$\mathrm {^3}$ and 0.30 mgHg/dm$\mathrm {^3}$, the amount of chlorophyll a and b decreased by about 60%. In the case of the chlorophyll a/b, the most deviations from the 3:1 value were observed for the cultured plants.

It should point out that, despite the increase in biomass and amount of protein under all experimental conditions tested (concentrations and contact time), the presence of mercury ions inhibited chlorophyll synthesis. The toxic effect could be a reduction in their concentration leading to chlorosis (Beauvais-Flück et al. , 2018).

**Fig. 8:**
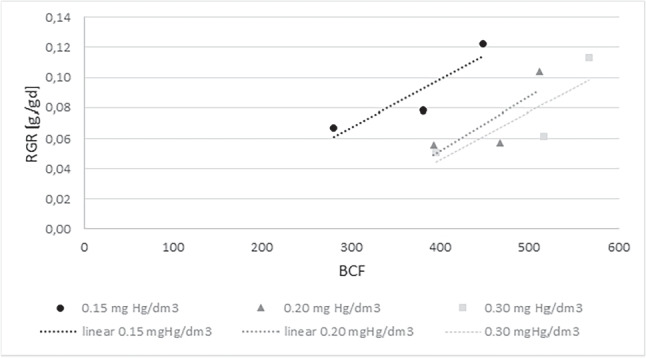
RGR dependence on BCF for cultured *Salvinia natans*

Figure 8 shows the dependence of RGR on BCF for the cultivated *S. natans*. Li and others’ study showed that the biomass of the plants showed a significant positive correlation with the first-order kinetic constant of toxic removal in the hydroponic system, indicating that plant biomass plays an important role in the remediation of contaminations (Li et al. , 2014). Analyzing the effect of the amount of accumulated mercury on the relative growth rate of the plants, we see a high correlation between the two parameters. As the amount of mercury in the substrate increased, the amount of mercury in the plants (accumulation) and the biomass growth rate increased. The linear correlation coefficient of the Persona bioaccumulation factor and Relative Growth Rate for a concentration of 0.15 mgHg/dm$\mathrm {^3}$ is 0.91, for 0.20 mgHg/dm$\mathrm {^3}$ it is 0.80, and for 0.30 mgHg/dm$\mathrm {^3}$ it took on a value of 0.82.

This gives a very high correlation for concentrations of 0.20 mgHg/dm$\mathrm {^3}$ and 0.30 mgHg/dm$\mathrm {^3}$. In the case of a concentration of 0.15 mgHg/dm$\mathrm {^3}$, an almost complete correlation.

**Fig. 9:**
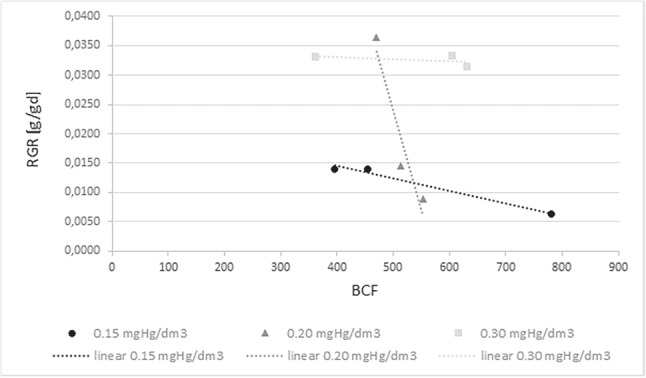
RGR dependence on BCF for *Salvinia natans* collected from the environmental

Also checked the dependence of RGR on BCF for plants taken from the environment (Fig. 9). In this case, an almost complete Pearson linear correlation was found for concentrations of 0.15 mgHg/dm$\mathrm {^3}$ ($-$0.99) and 0.20 mgHg/dm$\mathrm {^3}$ ($-$0.95). However, for a concentration of 0.30 mgHg/dm$\mathrm {^3}$, the correlation was 0.49, which is average. It should point out that for the breeding plants, that was a positive correlation, while for plants taken from the environment, it was a negative correlation.

Also analysed the relationships between the total chlorophyll and the mercury accumulated in the plants. Cultivated plants for concentrations of 0.15 mgHg/dm$\mathrm {^3}$ and 0.20 mgHg/dm$\mathrm {^3}$, showed a negative correlation of both parameters at ’weak’ and ’average’ levels. The concentration of 0.30 mgHg/dm$\mathrm {^3}$ observed a very high positive correlation. For environmental plants, the correlation of total chlorophyll and BCF was weak (0.15 - 0.30) for all three concentrations tested.

Analysing the relationship between total protein and accumulated mercury showed a high positive correlation for the concentration of 0.15 mgHg/dm$\mathrm {^3}$ in the cultured plants. For the other mercury concentrations in the medium, the correlation coefficient was close to -1. Also, plants taken from the environment obtained a correlation coefficient of low -1 for the concentration of 0.30 mgHg/dm$\mathrm {^3}$.

## Conclusion

As expected, despite the selection of relatively low mercury concentrations, an adverse effect of this element was observed on the parameters of the pleustophytes selected for the study. The performance of the phytoremediation process influences the physiological condition of the plants. The basic parameters based on which it is possible to assess their physiological condition are the content of total protein (the basic building component) and chlorophylls (assimilation pigments involved in the power of energy by plants). Total protein in the cultured *S. natans* decreased with increasing mercury concentration in the substrate but remained higher than for the control plants. This stimulation may indicate the production of proteins associated with the mercury detoxification process. The situation is quite different in the case of plants taken from the environment, where a reduction in the average amount of total protein in the plants was observed with the control samples. These were 429.96 mg/g DM (control), 403.30 mg/g DM (0.15 mgHg/dm$\mathrm {^3}$), 350.26 mg/g DM (0.20 mgHg/dm$\mathrm {^3}$) and 350.84 mg/g DM (0.30 mgHg/dm$\mathrm {^3}$). In the case of cultured *S. natans*, for all concentrations tested, a reduction in the average amount of assimilation pigments was observed compared to the values obtained for the control. Tests were also conducted for plants taken from the environment. Here, too, was achieved superimposition of stress on the toxic effect of mercury. Amounts of assimilation pigments and proteins confirm the disruption of physiological processes resulting in environmental stress and mercury toxicity. The RGR was in the range of 0.05$-$0.12 g/gd. The highest BFC was observed for plants taken from the environment. The removal of toxic metal was comparable for cultured and environmentally sampled plants. Mercury removal efficiency was 60-96%, depending on the amount of mercury in the medium and the type of plants used. An increase in the bioconcentration (BCF) of mercury in plants was observed during the first days of the experiments. In the later days of the research followed by a gradual decrease of mercury in the tissues. It could be the result of the released mercury in necrotic cell changes. A clear correlation between RGR and BCF was found, especially for farmed plants. No significant correlations of protein and chlorophyll amounts with BCF levels were.

Main conclusions:

- *S. natans* tolerates mercury concentrations up to 0.30 mgHg/dm$\mathrm {^3}$, so it can be used in the phytoremediation of mercury,
- Mercury significantly affects the physiological processes in plants, especially the biosynthesis of protein and assimilation of pigments and growth (biomass production). On the one hand, this confirms its toxicity, on the other hand, it indicates that the tested plants have a high degree of tolerance to mercury contamination.
- The results will expand knowledge on the phytoremediation of heavy metals (mercury) from the aquatic environment. Promising results, especially regarding the degree of metal accumulation in pleustophyte biomass, can be used in the design and operation of phytoremediation ponds or analogous solutions in wastewater treatment technology.

Phytoremediation technology is still not sufficiently understood and requires further research. Improving its efficiency by combining it with other methods, especially microbial methods, is still a new research subject. Also, emerging biomass is still a problem for this method. There are no methods for recovering mercury from biomass on an industrial scale.

## Data Availability

Not applicable.
